# A Novel Intelligent Fault Diagnosis Method for Rolling Bearings Based on Wasserstein Generative Adversarial Network and Convolutional Neural Network under Unbalanced Dataset

**DOI:** 10.3390/s21206754

**Published:** 2021-10-12

**Authors:** Hongtao Tang, Shengbo Gao, Lei Wang, Xixing Li, Bing Li, Shibao Pang

**Affiliations:** 1 Hubei Key Laboratory of Digital Manufacturing, School of Mechanical and Electronic Engineering, Wuhan University of Technology, Wuhan 430070, China; tanghongtaozc@163.com (H.T.); shengbo0419@whut.edu.cn (S.G.); libingjiayou2022@163.com (B.L.); pangshibao@foxmail.com (S.P.); 2 Hubei Key Laboratory of Modern Manufacturing and Quality Engineering, School of Mechanical Engineering, Hubei University of Technology, Wuhan 430068, China; li_xi_xing@126.com

**Keywords:** rolling bearings, intelligent fault diagnosis, data imbalance, generative adversarial networks (GAN), convolutional neural networks (CNN)

## Abstract

Rolling bearings are widely used in industrial manufacturing, and ensuring their stable and effective fault detection is a core requirement in the manufacturing process. However, it is a great challenge to achieve a highly accurate rolling bearing fault diagnosis because of the severe imbalance and distribution differences in fault data due to weak early fault features and interference from environmental noise. An intelligent fault diagnosis strategy for rolling bearings based on grayscale image transformation, a generative adversative network, and a convolutional neural network was proposed to solve this problem. First, the original vibration signal is converted into a grayscale image. Then more training samples are generated using GANs to solve severe imbalance and distribution differences in fault data. Finally, the rolling bearing condition detection and fault identification are carried out by using SECNN. The availability of the method is substantiated by experiments on datasets with different data imbalance ratios. In addition, the superiority of this diagnosis strategy is verified by comparing it with other mainstream intelligent diagnosis techniques. The experimental result demonstrates that this strategy can reach more than 99.6% recognition accuracy even under substantial environmental noise interference or changing working conditions and has good stability in the presence of a severe imbalance in fault data.

## 1. Introduction

Rolling bearings are widely used in industrial manufacturing. Ensuring the safe and stable operation of rolling bearings is the core requirement of the manufacturing process, and their health condition has a significant impact on system dependability, productivity, and facility lifetime [1,2,3]. In recent years, intelligent manufacturing engineering has become a significant development trend of the manufacturing industry, and the model-based mechanical fault diagnosis technology has been developed rapidly. A large number of methods and techniques have been proposed [4,5,6].

Due to its robust feature learning ability, deep learning has become the hot issue at present and provides new ideas for fault diagnosis of mechanical equipment [7,8,9,10]. Training the model to convergence requires vast quantities of labeled data for supervised learning in deep learning network models. It is a prerequisite to ensure that the number of samples between each category is balanced. The model learns balanced features under each data category to achieve high classification accuracy. However, in practical applications, there are severe imbalances and distribution differences in fault data, which lead to the incomplete training of deep learning networks and the inability to completely fit the distribution of training samples, ultimately leading to the poor classification accuracy of the model. Consequently, it is of great significance to establish a stable and valid diagnosis method under unbalanced samples.

To effectively improve diagnosis performance under unbalanced samples, many scholars have carried out research on this topic and obtained some remarkable results. Duan et al. based on a description of support vector data, developed a multi-classification fault diagnosis strategy to improve diagnostic accuracy [11]. Zhang et al. designed a new classification method for unbalance faults in permanent magnet synchronous motors based on a discrete wavelet transform [12]. Nevertheless, the classification accuracy cannot be significantly improved just by improving the classification method. Only by obtaining more simulated data from the original data can we find the root of the problem. In 2014, Goodfellow and Pouget-Abadie designed a new data enhancement method called a generative adversarial network (GAN), which can supplement the sample space with insufficient data by performing a model synthesis on a limited number of types of samples [13]. GANs are widely used for their outstanding application prospects, including signal processing, pattern recognition, and national security [14,15,16]. Meanwhile, due to GAN’s excellent data expansion capability, many models with different structures have been derived [17,18].

However, the continuous optimization and improvement of the GAN model structure does not completely address the problems of convergence difficulty and training instability. In 2017, Gulrajani and Ahmed designed a new generative adversarial network approach called the Wasserstein generative adversarial networks with gradient penalty (WGANs-GP) [19]. It does this by randomly interpolating between the real sample and the generated sample to guarantee that the transition area between the real sample and the generated sample meets the Lipschitz Constraint. Further research showed that WGAN-GP can overcome the drawbacks of the aforementioned methods, and the application performs well in the field of fault identification [20,21,22,23,24].

Due to the multiformity of rotating machine systems and the intricacy of sensing data, “weak” classical machine learning methods based on artificial feature selection are hard to provide accurate classification results for. Data-driven methods have received aggrandized attention from researchers because of the advantages of their fast and efficient processing of mechanical signals, reliable fault detection results, and their powerful capability of not relying on a large amount of a priori expert knowledge [25,26]. Deep confidence networks (DBNs) [27], recurrent neural networks (RNNs) [28], autoencoders [29], convolutional neural networks (CNNs) [30], and numerous other neural networks have been applied in fault diagnosis.

In recent years, CNNs have been widely used in fault diagnosis. CNNs can use a deeper extraction of fault features and significantly reduce the number of parameters while automatically and accurately obtaining the implied information in vibration signals in different states [31,32]. Janssens et al. introduced convolutional neural networks (CNNs) to the field of fault diagnosis and designed a feature learning model for condition monitoring based on CNN [33]. Zhang et al. explicitly applied the raw time signal as the input of a one-dimensional CNN to achieve fault classification [34]. Peng et al. proposed a residual learning-based one-dimensional CNN combined with the original vibration signal for bearing fault diagnosis under variable operating conditions [35]. At the same time, some researchers tried to implement fault identification from the perspective of image processing, to eliminate the influence of manual features, which provides a new idea for fault diagnosis. Li et al. proposed a method for a highly depth sensitive feature extraction and pattern recognition using STFT and CNN [36]. Ding et al. provided a new approach by using deep ConvNet to automatically learn multiscale features of wavelet packet energy (WPE)-generated images and use them for bearing fault diagnosis [37]. Wen et al. proposed a LeNet-5-based CNN for fault diagnosis [38]. Although the above CNN and image processing-based fault diagnosis methods have an outstanding preponderance in fault state identification. However, these methods extract spatial and channel information from local sensory regions without considering the weights of feature mapping, which generates redundant features to some extent and increases the computational cost while reducing the nonlinear fitting ability of the model to the fault features.

Recently, attention mechanisms in the computer realm have drawn several researchers’ attention by selectively reinforcing adequate information and reducing superfluous feature information to obtain better network performance [39,40]. The attentional (SE) mechanism adaptively recalibrates the feature response of a channel approach by explicitly modeling the interdependencies between channels, bringing significant performance gains with minimal additional computational cost. Hu et al. proposed the self-attentive convolutional neural network (SECNN) by adding a novel architectural unit squeeze and excitation [41]. Roy et al. demonstrated increasing segmentation accuracy by efficiently merging SE blocks into three state-of-the-art F-CNNs on three challenging benchmark datasets [42]. Feng et al. proposed a semi-supervised meta-learning with a squeeze and excitation attention network (SSMN) and demonstrated the usability and validity of the method with three bearing datasets [43]. Compared with convolutional neural networks (CNNs) and numerous other CNN variants, SECNN can improve the model’s resistance to imbalanced data and the nonlinear fitting ability to fault features, while the number of parameters and the model computation in the SECNN structure is relatively small.

To address the problem of limited rolling bearing fault samples and the unbalanced distribution of fault categories and to further realize efficient and high precision fault diagnosis, an intelligent fault diagnosis method based on grayscale image transformation, WGAN-GP, and SECNN is proposed. Firstly, the collected original vibration signals were converted into corresponding grayscale images to obtain 2D image samples that are easy to process by the model to extract image features and visualize different bearing states; then, adversarial training was performed using WGAN-GP to generate more new samples with similar distribution to the original samples; finally, the expanded sample data were input to a deep feature extraction model based on compressed excitation to automatically learn grayscale image features of different fault states, and selectively enhance functional feature mapping and reduce redundant features on the convolution channel to output recognition results. The experimental results show that the method has good robustness and generalization ability, and has excellent recognition performance under the fault class sample imbalance condition.

The superiority and innovativeness of the method proposed in this study is summarized as follows:

1. The conversion of a one-dimensional original vibration signal to the two-dimensional grayscale image was realized by using grayscale image conversion technology to fully exploit the deeper feature information and better utilize the image generation capability of WGAN-GP;
2. A data-driven approach based on WGAN-GP was used to generate data samples with imbalanced bearing failure classes. Compared with GAN and WGAN, the WGAN-GP can solve the problems in GAN due to *JS* dispersion that leads to the WGAN-GP solving the problems of unstable GAN training and pattern collapse due to *JS* scatter, and the problems of neural network learning become simple function mapping, gradient disappearance, and gradient explosion due to the weight cropping implementation in WGAN. The choice of applying WGAN-GP to force the discriminator to satisfy the continuity constraint of the 1-Lipschitz function by adding a gradient penalty term results in faster convergence and better quality of generated samples;
3. The attention mechanism was introduced into the field of bearing fault diagnosis, and the self-attentive convolutional neural network (SECNN) was constructed, which can automatically extract information related to deep fault features and further improve the anti-interference ability and classification accuracy of the model for unbalanced data;
4. This method has outstanding performances in domain adaptation and can gain satisfactory diagnostic performance even when the working environment changes or the environmental noise is strong.

The method has a strong domain adaptive capability. The organizational framework of this paper is illustrated as follows. Section 2 introduces the essential theoretical background of CNN, GANS, and signal-to-image converting methods. In the Section 3, the proposed intelligent fault diagnosis framework is described in detail. In the Section 4, the availability and superiority of this method are verified by experiments, and the experimental results are compared with other deep learning models. In the Section 5, conclusions and future work are summarized.

## 2. Theoretical Background

### 2.1. Signal to Image Converting Method

Due to the ineffectiveness of generative adversarial networks indirectly processing 1D time-domain signals and the formidable feature extraction ability of convolutional neural networks for image data, the raw data need to be preprocessed [44,45]. In this paper, a preprocessing method of grayscale image conversion is proposed, which converts the collected one-dimensional time-domain signals into two-dimensional grayscale images to reduce the noise impact on fault classification accuracy and fully exploit the fault features in the data. The conversion method consists of the following three main steps.

#### 2.1.1. Signal Interception Using a Sliding Window

**Figure 1:**
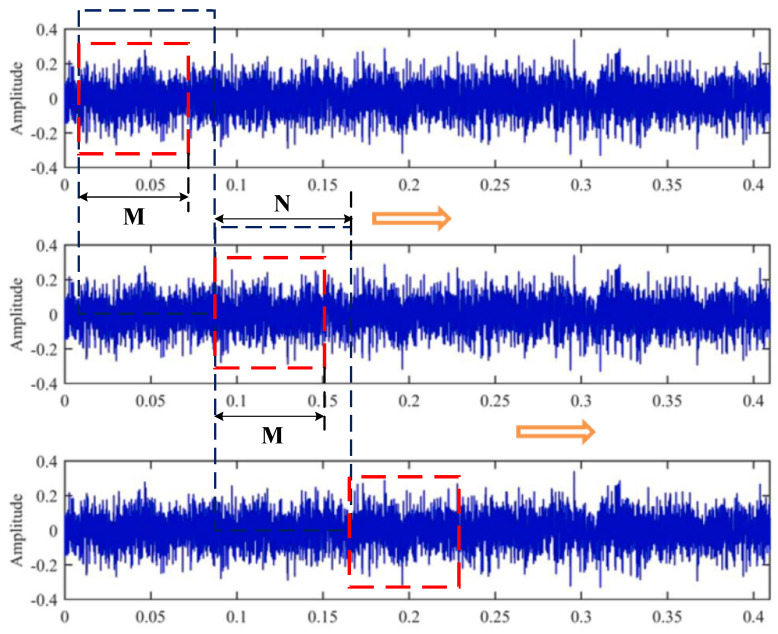
Schematic diagram of the sliding window fetching method.

For a one-dimensional time-domain signal, the signal was first intercepted through a sliding window. As shown in Figure 1, the size of the window is *M*, which means that *M* units of data were taken at each time, where it must be ensured that each *M* contains at least one complete cycle of data. After each fetch, the window was moved backward by *N* units.

#### 2.1.2. Data to Image Conversion

**Figure 2:**
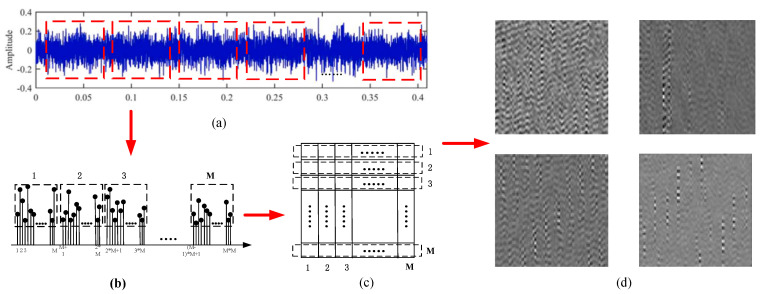
Signal to image conversion method. (**a**) Signal Interception Using a Sliding Window, (**b**) Combination of Vibration Signal Sequences, (**c**) Grayscale Image Transformation, (**d**) Grayscale Image.

The signal-to-image conversion process is shown in Figure 2. First, *M* sub-signal columns of length *M* were selected using the sliding window fetching method; the randomly selected sub-signal columns were combined to obtain a signal column of length *M* × *M*. The mixed signals were converted into grayscale images using Equation (1).

(1) $$P\left(j,k\right)\;=\;round\;\left\{\frac{L\left(\left(j-1\right)\times M+k\right)-Min\left(L\right)}{Max\left(L\right)-Min\left(L\right)}\times 255\right\}$$

where round{.} denotes the rounding function, which normalizes all pixel values to between 0 and 255, exactly the pixel value domain of the grayscale image, $P\left(j,k\right)$, *j* = 1, …, *M*; *k* = 1, …, *M* denotes the pixel value of each image after transformation and *M* represents the size of the grayscale image, $L\left(i\right)$, *i* = 1, 2, …, *M* denotes the pixel intensity of each image.

#### 2.1.3. Batch Processing Operation of Images

To ensure that the gradient of the network descends toward the lowest point, the convergence of the network was sped up, the amount of computation was reduced, and memory overflow was prevented. The images were batch-processed by dividing the entire sample data into different batches, each batch containing the same amount of data *M*. Each training was performed on one batch, and the direction of gradient descent was determined by the average gradient of all batches of data in that batch.

In the process of transforming the 1D time-domain signal into a 2D grayscale image, all data implicit in the image were preserved. The noise in the data was transformed into grayscale, luminance, and other information in the image that is not relevant to the image classification result, thus minimizing the impact of signal noise on the classification accuracy and achieving the purpose of noise reduction. At the same time, the transformation process is an end-to-end process that does not require any human expert intervention at all, and the whole input-output process is entirely completed by the transformation formula, further promoting the application of automation in intelligent manufacturing.

### 2.2. Generative Adversarial Nets (GANs)

**Figure 3:**
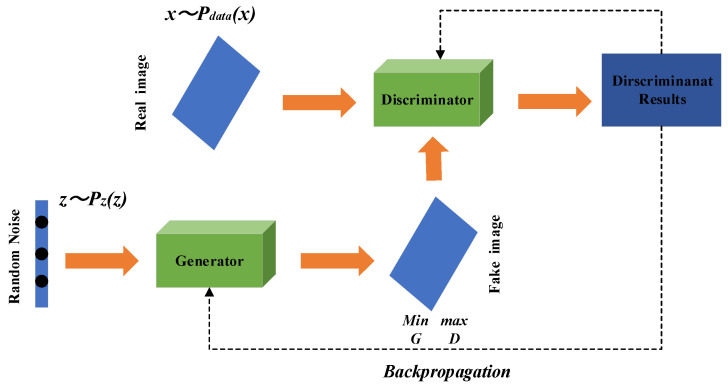
The structure of a GAN.

GAN is a generative model based on game theory. The GAN model mainly consists of two independent sub-modules, the generator and the discriminator, as shown in Figure 3. During the model training process, the generator generates a simulated signal confusing the discriminator by fitting data features and adding noise randomly, and the purpose of *D* is to maximize the identification of the input data as the simulated signal *G*(*z*) generated by *G* and the data *x* in the original dataset. By continuously self-optimizing through adversarial training to improve their generative and discriminative abilities, it is ultimately the objective function that reaches an optimal solution, i.e., a Nash equilibrium between *G* and *D* [18,46]. The objective training function of the GAN model is shown below:

(2) $$\underset{G}{min}\underset{D}{max}V(D,G)=E_{x∼p_{\mathrm{data}}\left(x\right)}\left[\log D\left(x\right)\right]+E_{z∼p_{z}\left(z\right)}\left[\log\left(1-D\left(G\left(z\right)\right)\right)\right]$$

where *z* denotes the random noise vector from the prior distribution $P_{z}\left(z\right)$ and *x* is the real input data with distribution $P_{\mathrm{data}}\left(x\right)$. $D\left(x\right)$ denotes the output result of the discriminator *D*, where the *sigmoid* function is used as the activation function (AF). When the output result $D\left(x\right)$ > 0.5, *D* treats the input *x* as a true sample and vice versa, *D* treats the input *x* as a false sample.

Equation (2) can be split into two parts: maximizing *D* and minimizing *G*, as shown in Equations (3) and (4) below.

(3) $$\underset{D}{max}{\;E}_{x∼p_{\mathrm{data}\;}\left(x\right)}\left[\log D\left(x\right)\right]+E_{x∼p_{g}\left(x\right)}\left[\log\left(1-D\left(x\right)\right)\right]$$

(4) $$\underset{G}{min}\;E_{x∼p_{x}\left(x\right)}\left[\log\left(1-D\left(x\right)\right)\right]$$

The literature [13] proved that the optimal discriminator *D* is (5) when the generator *G* is fixed:

(5) $$D_{G}^{*}\left(x\right)=\frac{p_{\mathrm{data}\;}\left(x\right)}{p_{\mathrm{data}\;}\left(x\right)\;+\;p_{g}\left(x\right)}$$

Equation (5) reaches its optimality when $P_{g}=P_{\mathrm{data}}$, a Nash equilibrium is reached. At this point, Equation (4) can be transformed into Equation (6): $\underset{G}{min\;}$ can be converted into:

(6) $$2\cdot JSD\left(p_{\mathrm{data}\;}∥p_{g}\right)-2\log\left(2\right)$$

*JSD* is the Jensen–Shannon scatter, and is used to compare the discrepancy between $P_{x}\;$ and $P_{g}$. Therefore, the optimization process of GAN can be regarded as the continuous optimization process of the *JS* divergence between the generated samples and the real samples. In other words, when the *JS* divergence becomes zero, the model has the optimal performance.

The Wasserstein generative adversarial network (WGAN) analyzes the causes of the *JS* scatter at the theoretical level, effectively solves the problem, and guarantees the diversity of the generated samples. The Wasserstein generative adversarial net (WGAN) evaluates the difference between the real and generated sample distributions by using the Wasserstein distance, which has soothing properties superior to Jensen–Shannon scattering.

Through a mathematical transformation, the Wasserstein distance can be transformed into the following solvable form, as shown in Equation (7):

(7) $$W\left(P_{r},P_{\theta }\right)\;=\;\underset{\gamma ∼\prod \left(P_{r},P_{\theta }\right)}{inf}E_{\left(x,y\right)∼\gamma }\left[∥x-y∥\right]$$

where $P_{r}$ and $P_{\theta }$ are the distributions of the primeval and simulate data, $\prod \left(P_{r},P_{\theta }\right)$ represents the joint distribution, $\left(x,y\right)$ samples from the joint distribution γ, $E_{\left(x,y\right)∼\gamma }\left[∥x-y∥\right]$ represents the expectation of the distance, inf is the lower boundary of the set, and $W\left(P_{r},P_{\theta }\right)$ is the Wasserstein distance of the distributions $P_{r}$,$P_{\theta }$.

The objective function of WGAN is shown as follows:

(8) $$\underset{G}{min}\underset{D\in \mathbb{R}}{max}E_{x∼p_{x}}\left[D\left(x\right)\right]-E_{y-p_{g}}\left[D\left(y\right)\right]$$

where ℝ is the set of 1-Lipschitz functions. The Lipschitz limit is achieved by adding the upper bound so that the output value given by the discriminator does not change much when the input sample fluctuates slightly.

However, there are still two problems with the implementation of WGAN weight cropping that make the optimization process difficult. First, most of the weights of the network are concentrated at both ends, which makes the learning of neural networks easy to become simple function mapping. The powerful fitting ability of WGAN cannot be fully exploited. Second, the forced cropping of the network weights tends to cause gradient disappearance or gradient explosion. The above problems can be entirely solved by WGAN-GP, by employing the addition of gradient penalty terms to force the discriminator to satisfy the continuity constraints of the 1-Lipschitz function.

The loss function of the generator *G* is kept constant, and the loss function of the discriminator *D* is shown below:

(9) $$L\left(D\right)\;=\;\underset{\mathrm{Originalloss}\;}{\underbrace{E_{x∼p_{x}}\left[D\left(x\right)\right]-E_{z∼p_{z}}\left[D\left(G\left(z\right)\right)\right]}}+\underset{\mathrm{Gradient}\;\mathrm{penalty}}{\underbrace{\lambda E_{\hat{x}∼p_{\hat{x}}}{\left[∥\nabla_{\hat{x}}D{\left(\hat{x}\right)∥}_{2}-1\right]}^{2}}}$$

where $P_{\hat{x}}$ is the distribution of $\hat{x}$, $∥\nabla_{\hat{x}}D\left(\hat{x}\right)∥$ denotes the discriminant gradient. Adding a gradient penalty to WGAN makes the model have more stable gradients, which neither disappear nor explode, and which converge faster, and generate samples with better quality.

### 2.3. Convolutional Neural Network (CNN)

CNNs are feedforward neural networks consisting of multiple convolutional and pooling operations with excellent automatic feature extraction capability and can handle overfitting problems, which have shown remarkable performance in areas such as image processing, pattern recognition, and target tracking.

**Figure 4:**
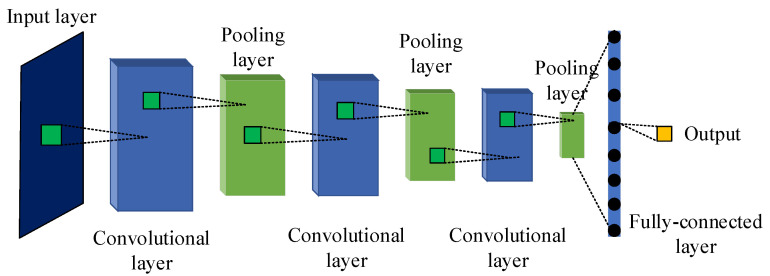
The classical structure of CNN networks.

**Figure 5:**
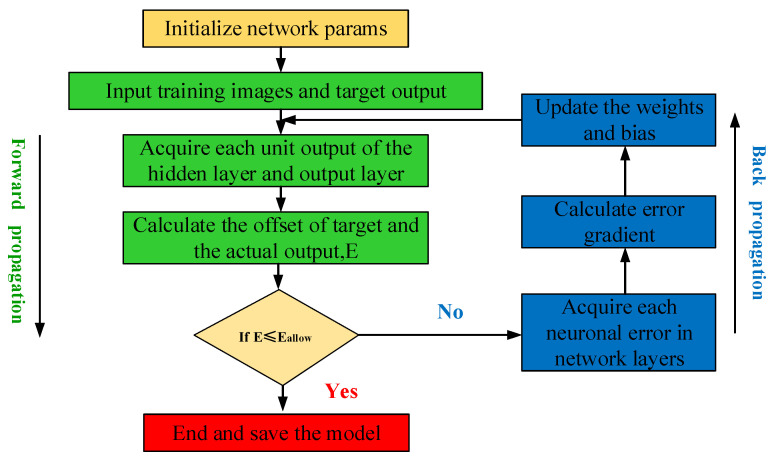
The training process of CNN.

The typical structure of CNN networks is shown in Figure 4. The training process of CNNs mainly consists of a forward propagation process and reverse parameter update, as shown in Figure 5.

#### 2.3.1. Forward Propagation Process

(a) Convolutional layer: CNN performs the convolutional operation on the original input image by convolutional kernels of different sizes. The convolution operation formula is shown below:

(10) $$x_{j}^{l}=f\left(\sum_{i\in M_{j}}x_{i}^{l-1}*w_{ij}^{l}+b_{j}^{l}\right)$$

where $x_{j}^{l}\;$ denotes the jth element of the lth layer, $M_{j}\;$ denotes the jth convolutional region of the l−1th layer feature mapping, $x_{i}^{l-1}$ is denotes the element in the l−1th layer, $w_{ij}^{l}$ is the weight matrix of the lth layer, $b_{j\;}^{l}$ is the deviation, and f is usually the nonlinear *ReLU* activation function. The operator formula of the nonlinear *ReLU* activation function is:

(11) $$f\left(x\right)\;=\;max\left(0,x\right)$$

(b) Pooling layer: In the pooling layer, redundant features are reduced by downsampling to improve the nonlinear fitting ability to the fault features and reduce the network parameters and computational cost. The pooling operation can be expressed as:

(12) $$x_{j}^{l}=f\left(\beta_{j}^{l}*\mathrm{down}\left(x_{j}^{l-1}\right)\;+\;b_{j}^{l}\right)$$

where $\beta_{j}^{l}$ and $b_{j}^{l}$ denote the weight and deviation of the jth feature map in the lth layer, respectively, down( ) is the down-sampling function.

(c) Fully connected layer: After several alternating operations of convolution and pooling, the sample classes and probabilities can be input on the fully connected layer. The operation formula of the fully connected layer can be expressed as:

(13) $$y^{k}=f\left(w^{k}x^{k-1}+b^{k}\right)$$

where k denotes the numerical order of the network layers, $y^{k}$ denotes the output of the fully connected layer, $x^{k-1}$ denotes the unfolded 1D feature vector, $w^{k}$ is the weighting factor, $b^{k}$ denotes the bias.

#### 2.3.2. Backpropagation of Parameter Updates

Updating parameters by forwarding propagation alone cannot guarantee the recognition accuracy of the model, and it is necessary to update network parameters in reverse. This model used the classification cross-entropy loss function, and its expression is as follows:

(14) $$E=\frac{1}{n}\sum_{k=1}^{n}\left[y_{k}\ln t_{k}+\left(1-y_{k}\right)\ln\left(1-t_{k}\right)\right]$$

where n is the sample size, $y_{k}$ and $t_{k}$ are the actual objective and predicted values of the sample, respectively. The gradient descent method is used to minimize the loss function, and then the partial derivatives are calculated by Equation (15) to gradually update the adaptive parameters w and b.

(15) $$\begin{array}{c} w_{ij}^{l}|=w_{ij}^{l}-\alpha \frac{\partial E}{\partial w_{ij}^{l}}\\ b_{j}^{l}|=b_{j}^{l}-\alpha \frac{\partial E}{\partial b_{j}^{l}} \end{array}$$

where α is the learning rate that controls the parameter update step. In this paper, we use a time-based learning progress schedule with the following expressions:

(16) $$\alpha =\alpha *1/\left(1+\;decay\;*\;epoch\right)$$

where, *decay* indicates that the learning rate is reduced from a given fixed value from the previous period, and *epoch* represents the current training period.

#### 2.3.3. Squeeze and Excitation CNN

Convolutional neural networks extract spatial and channel information through local perceptual areas, but do not consider the weights of feature mappings within disparate convolutional channels, generating redundant features to some extent and making the model less capable of fitting nonlinearities to faulty features. Recently, attention mechanisms in the computer realm have drawn several researchers’ attention by selectively reinforcing adequate information and reducing superfluous feature information to obtain better network performance. Therefore, to further promote the implementation of convolutional neural network models, we fuse CNN models with attention mechanisms.

Squeeze and excitation (SE) is a novel CNN attention mechanism proposed by Hu et al. [41], which is applied to image classification to improve the performance of image representation significantly. The SE mechanism can characterize more information with a minimum number of parameters and assign a weight to each channel based on global information. It has two major components, the squeeze operations and the excitation operations.

The squeeze operation generates channel descriptors by integrating global spatial information, where each element corresponds to a feature map information. In the stimulation operation, channel statistics are taken as input, and the descriptors are adapted to determine the attention weights for each channel through two fully connected layers. Finally, the consequences are used to adaptively recalibrate the feature map, through which the feature map model can emphasize useful information.

**Figure 6:**
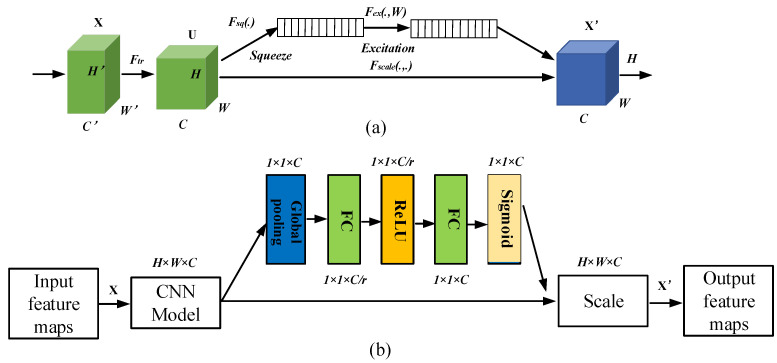
(**a**) SE network. (**b**) SECNN module.

For a given input of size $\left(H^{\prime },W^{\prime },C^{\prime }\right)$, mapped into a feature map *U* ($U\in R^{W\times H\times C}$), where *R* denotes the set of real numbers, *W* and *H* denote the width and height of the feature map, and *C* denotes the number of feature maps. The SE network is shown in Figure 6.

The given input $\left(H^{\prime },W^{\prime },C^{\prime }\right)$ is first mapped to the feature map $U=\left[u_{1},\;u_{2},\;\ldots ,u_{c}\right]$ by a series of convolutional transformations $F_{tr}$ in the following equation:

(17) $$U_{RPC}=F_{C}\left(X_{RPC}\right)\;=\;\sum_{i=n}^{C^{\prime }}v_{i}^{*}x_{RPCi}$$

where $U_{RPC}$ denotes the set of local descriptors whose statistics represent all influences of the *RPC*, $U_{RPC}\in R^{W\times H}$. $\;X_{RPC}$ denotes the influence factor of the *RPC* or the output of the uppermost maximum pooling layer of the *RPC*, $X_{RPC}\in R^{W\times H\times C}$. $v_{i}$ denotes the 2D convolution kernel, and * denotes the convolution operation.

Since $U_{RPC}$ is generated by summing all channels, channel correlations are implicitly intertwined in $v_{i}$ with the spatial correlations captured by the filter. The squeeze operation aims to squeeze the feature information into the channel descriptors. This is achieved by using a global averaging pool, the compression transformation $F_{s}$ to map the feature $U_{RPC}$ to a global spatial one-dimensional feature vector, transforming each two-dimensional feature channel into a statistic $S\in R^{C}$.

(18) $$S=F_{S}\left(U_{RPC}\right)\;=\;\frac{1}{H\times W}\sum_{i=1}^{H}\sum_{j=1}^{W}U_{RPCi,j}$$

Then, a stimulus operation is executed. A weight evaluation is performed for each channel of the adaptive feature recalibration through a self-gating mechanism containing two fully connected layers. $W_{1}$ is used for dimensionality reduction and $W_{2}$ is the opposite of $W_{1}$. $E\in R^{C}$ reflects the criticality of each feature channel.

(19) $$E=F_{E}\left(S,W\right)\;=\;Sigmoid\left(W_{2}\cdot ReLU\left(W_{1}\cdot S\right)\right)$$

where *Sigmoid*( ) and *ReLU*( ) are the two activation functions, $W_{1}\in R^{\left(C/r\right)\times C}$ and $W_{2}\in R^{C\times \left(C/r\right)}$, and *R* express the dimensionality reduction ratio. The redundant channel information is suppressed by the dimensionality reduction weight matrix $W_{1}$, and the excitation matrix *E* is adjusted to map the dimensionality of $U_{RPC}$ using the dimensionality increase weight matrix $W_{2}$. Finally, the output is used as the weight of each feature channel and reweighted:

(20) $${\tilde{X}}_{RPC}=F_{\mathrm{Scale}}\;\left(U_{RPC},E\right)\;=\;E\cdot U_{RPC}$$

where ${\tilde{X}}_{RPC}\in R^{W\times H\times C}$ is the final output of SE. $F_{\mathrm{Scale}}$ denotes the product between convolutional features,$\;U_{RPC}$, and channel weights. The detailed implementation process of the SECNN module is provided in Figure 6b.

## 3. The Proposed Joint Method

### 3.1. Diagnosis Framework

It is a great challenge to train a deep model with millions of parameters for accurate fault diagnosis due to the limited training data in fault states and unbalanced fault data classes. Based on the signal to image conversion technique, GANs, and convolutional neural networks mentioned earlier, this section proposes a new bearing fault diagnosis framework based on the signal to image conversion technique, WGAN-GP, and SECNN models.

**Figure 7:**
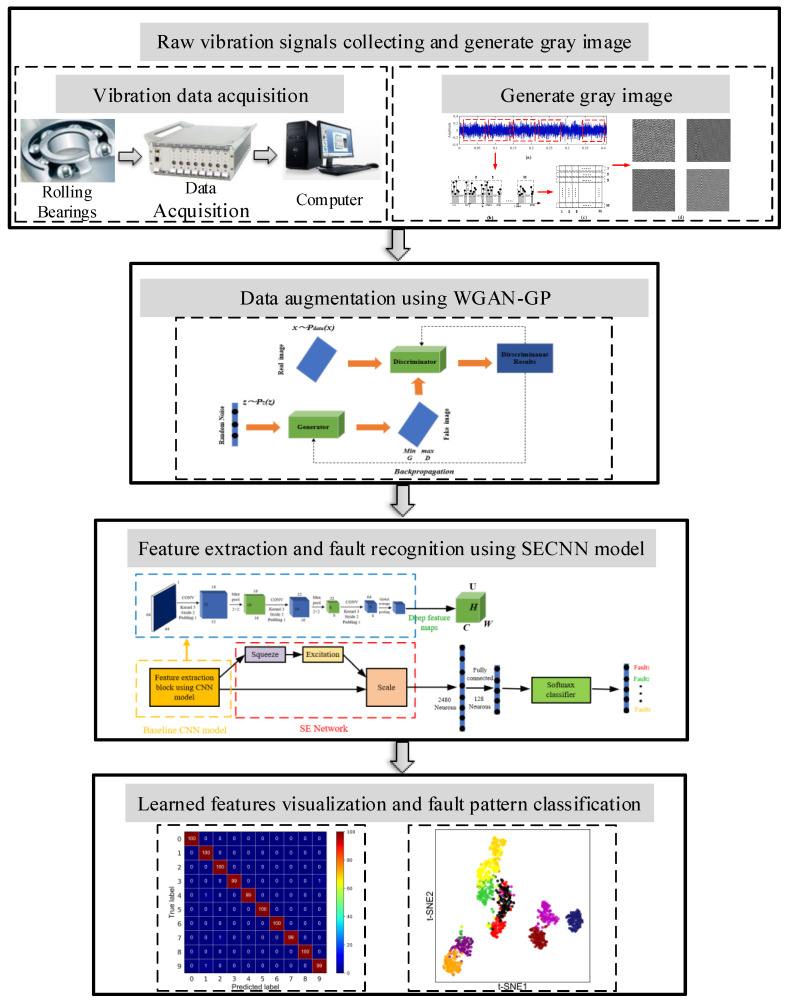
The diagnostic framework based on WGAN-GP and SECNN.

It takes full advantage of the image conversion technique in the feature extraction field, CNN in the image recognition field, and GANs in the sample generation field. The detailed fault diagnosis framework is shown in Figure 7. This fault diagnosis framework has four main steps: raw vibration signals collecting and generating the grey image, data augmentation using WGAN-GP, feature extraction and fault recognition using the SECNN model and learned features visualization, and fault pattern classification.

### 3.2. Bearing Fault Diagnosis Flow

**Figure 8:**
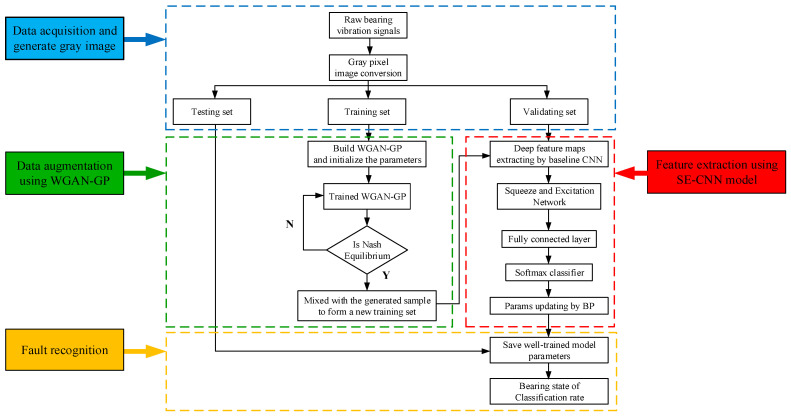
Flowchart of WGAN-GP and SECNN implementation.

The whole rolling bearing intelligent fault diagnosis flow chart is shown in Figure 8. It consists of the following four processes: (1) data acquisition and generating a grey image; (2) data augmentation using WGAN-GP; (3) feature extraction using the SECNN model; (4) and fault recognition. The detailed fault diagnosis steps are as follows:

1. The 1D time-domain vibration signal acquired by the acceleration sensor is converted into a 2D grayscale image with pixel values ranging from 0 to 255 and a size of 64 × 64 using grayscale image conversion techniques;
2. The data are randomly partitioned into training dataset, test dataset, and validation dataset according to the corresponding fault states and scales;
3. Each type of training sample is input into the WGAN-GP model for adversarial training until Nash equilibrium is reached, while new samples are integrated into the original training set to expand the training set;
4. The new training set is fed into the established SECNN for training, and the *Softmax* classifier is used to identify the fault states and their classes;
5. Finally, the trained neural network model is tested with test samples, and the results of fault detection are output to assess the diagnostic performance of the method.

## 4. Experimental Validation

In this section, to evaluate and validate the performance of the constructed fault diagnosis framework and the validity of the proposed algorithm, we experimentally compared the popular CNNs and analyzed the robustness and generalization capability of the method in bearing imbalance fault diagnosis for the measured vibration signals of rolling bearings. The operating environment of the algorithm is 2.7 GHz CPU, 8 GB main memory, NVIDIA GeForce GTX 1060 3 GB GPU; the programming environment is Python 3.8.3.

### 4.1. Dataset Description

**Figure 9:**
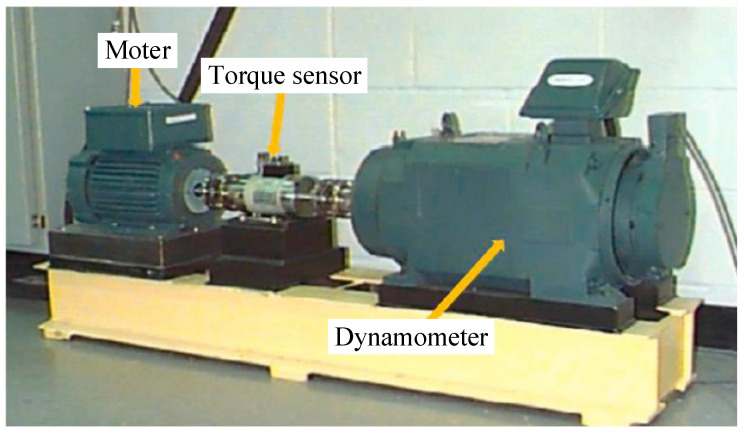
The rolling bearing fault simulation test platform.

The case data are rolling bearing benchmark data acquired from the Case Western Reserve University (CWRU) Bearing Data Center. The simulated test terrace of CWRU is shown in Figure 9. The rolling bearing to be tested is a 6205-2RS JEM SKF deep groove ball bearing, and the detailed parameters of this rolling bearing are listed in Table 1.

The test motor was operated at 1730 r/min, and the bearing health and fault data at the drive end were sampled at a frequency of 12 k. The CWRU dataset contains four different status categories: normal (N), outer race fault (OF), inner race fault (IF), and ball fault (BF). There are 3 different failure sizes for each failure condition: 0.007 in. (0.1778 mm), 0.014 in. (0.3556 mm) and 0.021 in. (0.5334 mm). Therefore, a total of 10 operating states were set up for this experiment, and the specific classification is shown in Table 2.

Above all, the time-domain signal collected by the acceleration sensor was decomposed into multiple fragments for sample generation. The length *M* of the fragments was set to 64, considering the computational performance and preventing memory overflow, and then they were converted into grayscale images with pixel values ranging from 0 to 255 and a size of 64 × 64. To confirm the diagnostic precision of the proposed method, we selected the same proportion of data from the nine rolling bearing fault datasets described in Table 2 for experiments.

The division of the datasets and the number of samples in each sub-dataset are shown in Table 3. Dataset *A* represents the raw dataset, *B* is the training dataset stochastic selected at 60% from the original dataset *A*, *C* is the test dataset chosen randomly at 40%, *D* is the generated dataset of WGAN-GP, and dataset *E* is the enhanced dataset formed by combining *B* and *D*. During the training process, 15% of the dataset *A* were used to verify the precision of the proposed method to adjust hyperparameters.

### 4.2. Enhancement Data and Accuracy

In this section, we first estimated the effectiveness of WGAN-GP in generating and extending data to address the severe data imbalance and distribution discrepancies in a limited data fault diagnosis. To maximize the effectiveness of WGAN-GP data generation, we determined the value of the gradient penalty factor λ through comparison experiments for subsequent experiments. As shown in Table 4, In order to minimize particularity and contingency, each experiment was repeated ten times, and the average result of the ten experiment results was regarded as the accuracy of the model. When the gradient penalty factor λ is set to 10, the experimental results have high accuracy.

Second, to precisely contrast the sample generation effect of GAN, WGAN, and WGAN-GP, we used the Fréchet distance (F) as a measurement. The experimental and computational results comparison are shown in Table 5, so the sample generation ability of WGAN-GP is more substantial, and the similarity is higher.

**Figure 10:**
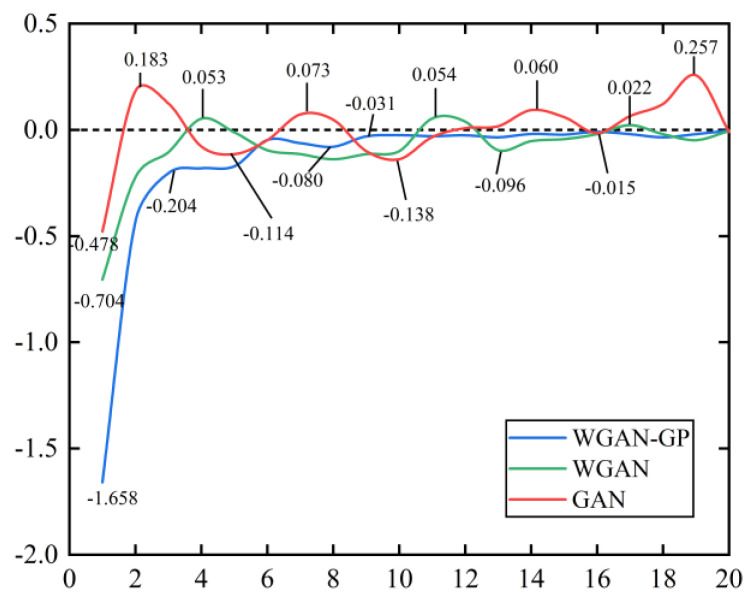
The generator loss function value change curve.

**Figure 11:**
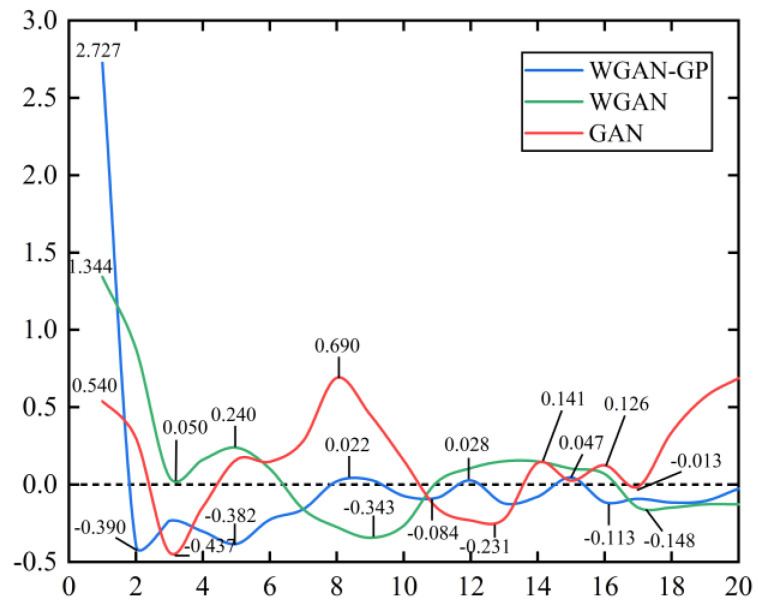
The discriminator loss function value change curve.

The change curves of the loss function values of the WGAN-GP model are shown in Figure 10 and Figure 11, where the data values are taken once every 5000 iterations for a total of 20 loss functions values. During 100,000 iterations, the loss function values in all three GANs models exhibited large oscillations in the early phase and are more stable in the middle and later periods. It is evident that the WGAN-GP model is much more stable than GAN and WGAN in the middle and late stages, and the loss function values keep converging to zero.

During the WGAN-GP generation of sample data, the WGAN-GP model was trained to form a Nash equilibrium between the generator and discriminator. The L2 regularization penalty was set to 1 × 10^−5^ in the discriminator, and the Adam optimizer was used for both the generator and the discriminator.

To promote the diagnostic performance and the nonlinear fitting ability of the SECNN model to the fault features under the unbalanced sample condition, we divided it into nine experimental groups for comparison experiments by setting the number of convolutional kernels and activation functions in each convolutional layer differently. From Table 6, it can be seen that optimal identification precision is achieved when the number of convolutional kernels in convolutional layers is 16, 32, and 64, respectively, and the type of activation function is *Leaky ReLU*.

**Figure 12:**
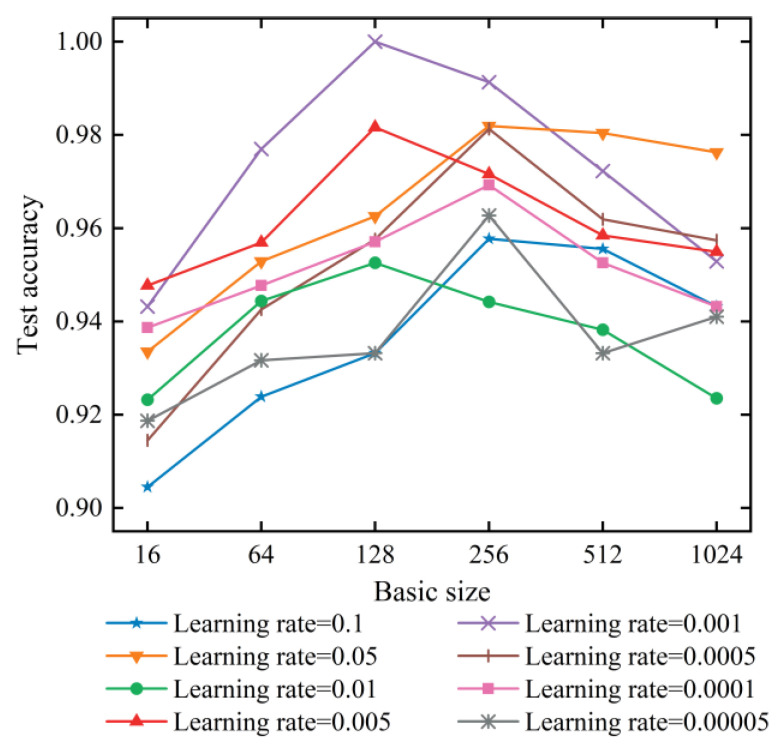
Comparison of batch size experiment and learning rate size.

**Figure 13:**
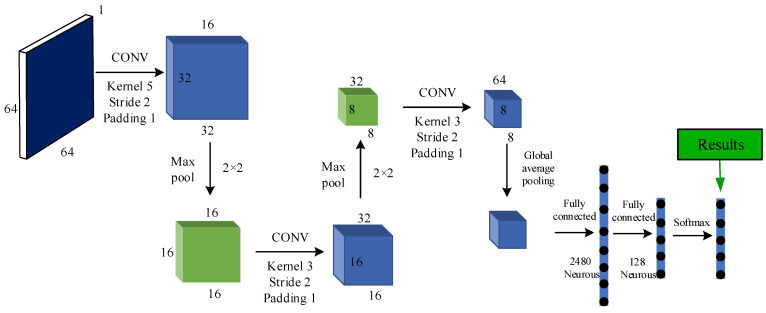
A specific architecture of SECNN.

The experiments analyzed the effects of batch size and learning rate on fault diagnosis accuracy. From Figure 12, it can be seen that the highest identification precision is achieved when the batch size and learning rate are set to 128 and 0.001, respectively. The dimensionality reduction rate r of the SE module was set to 8. Therefore, we set this structural parameter in all subsequent experiments. The specific architecture of SECNN is shown in Figure 13.

We also defined the algorithm efficiency factor λ to maximize the model diagnostic performance. The calculation formula is shown in Equation (21). We performed five sets of comparison experiments for the number of training iterations of the selected model, and the comparison of the experimental and computational results are shown in Table 7. Through the comparison experiments, we found that set iterations to 100,000 can obtain more satisfactory results.

(21) $$\lambda =\frac{\mathrm{Test}\;\mathrm{accuracy}\;}{\mathrm{Total}\;\mathrm{time}\;}*100$$

### 4.3. Diagnosis Accuracy Comparisons

In this section, to further verify the validity of the proposed rolling bearing diagnosis strategy, we explored the diagnostic performance of different data mining algorithms by setting up comparative experiments. dataset *C*, with 40% of samples randomly selected in the original dataset was fed into other deep learning models.

To minimize the specificity and chance of the experimental results, we repeated each experiment ten times with the same dataset. A proposed paper comparing the algorithmic models in References [38,47,48,49,50,51,52,53] is provided. As can be seen from Table 8, the average accuracy of all models for the unbalanced dataset exceeds 70%, but there is a large variability in the diagnostic results between different models under the same dataset.

From the comparison results, it can be seen that the original CNN model has the lowest identification precision of 72.40%. At the same time, the diagnosis accuracy is improved for SECNN with the addition of the self-attention mechanism, which indicates that the self-attention module has a more prominent role in suppressing the noise weight and enhancing the weight of fault features. Both algorithm GAN-SECNN and algorithm WGAN-GP + SECNN are fault diagnosis methods based on generative adversarial networks, and the classification accuracy of the WGAN-GP + SECNN algorithm is 100%, which is higher than that of GAN-SECNN. Its diagnostic accuracy is greatly improved compared with that of SECNN-based fault diagnosis methods, which indicates that generative adversarial networks can cope well with unbalanced data and significantly reduces the reliance on raw data while considering the diagnostic accuracy, which has a more significant advantage over other mainstream fault diagnosis methods.

**Figure 14:**
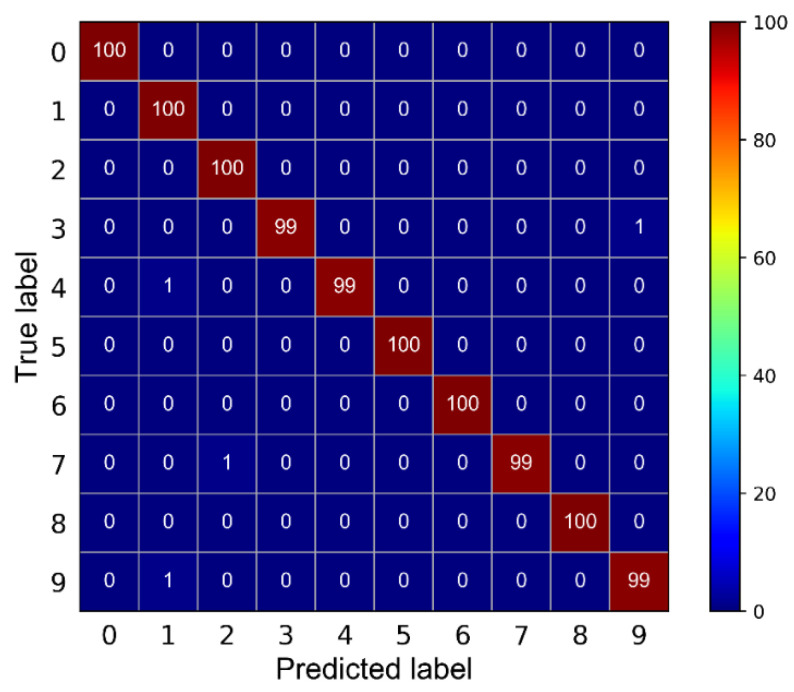
Multi-class confusion matrix of the presented method.

Second, we input dataset *B* as the training set into the proposed model and dataset *C* as the testing set. The confusion matrix was introduced to show more directly the accuracy of the proposed model for identifying the various fault states of rolling bearings. Figure 14 shows the confusion matrix of the results. The experimental results show that the model can reach fast convergence and high diagnostic accuracy under data imbalance.

**Figure 15:**
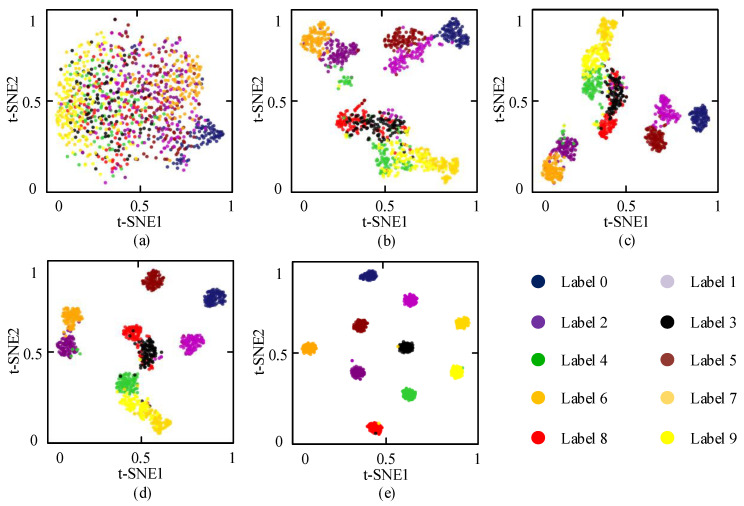
Feature visualization via t-SNE: (**a**) original signal; (**b**) conv layer1; (**c**) conv layer2; (**d**) conv layer3; (**e**) *FC layer*.

To visualize the feature extraction capability of the WGAN-GP + SECNN model, t-SNE was used to map extracted high-dimensional features to a two-dimensional space, as shown in Figure 15.

From Figure 15a, we can observe that when the original features in the test set are transformed into two dimensions by t-SNE, various fault states are overlapped, making it almost impossible to distinguish the boundaries between the categories. With the increasing number of iterations, the points of the same category are gradually clustered, but it is still difficult to distinction all the categories, as shown in Figure 15b–d. Finally, sample points with the same color are clustered together, and each fault boundary under ten working conditions can be distinguished, as shown in Figure 15e. The feature visualization results show that the WGAN-GP + SECNN model can reach identification precision accurately.

### 4.4. Generalization and Robustness Comparisons

In the actual rolling bearing fault diagnosis process, from time to time, we faced changes in the working conditions, resulting in large distribution differences between the training data and the test data, which makes the fault diagnosis performance degraded. To confirm the generalization ability and robustness, fault diagnosis experiments were conducted for rolling bearings under different working conditions.

In this part of the experiments, each dataset is a multi-speed mixed dataset. The training and testing samples in dataset *A*_1_ are composed of the same data from loads of 0–3 hp, the training and testing samples in dataset *B*_1_ are composed of different data from loads of 0–2 hp and the load of 3 hp, the training and testing samples in dataset *C*_1_ are composed of different data from loads of 0–1 hp and the load of 2 hp, and the crack size was added to dataset *D*_1_ variables. The detailed dataset distribution is shown in Table 9. The generalization ability and robustness of the proposed model were evaluated by conducting experiments under the same parameter settings as in the previous experiments.

**Figure 16:**
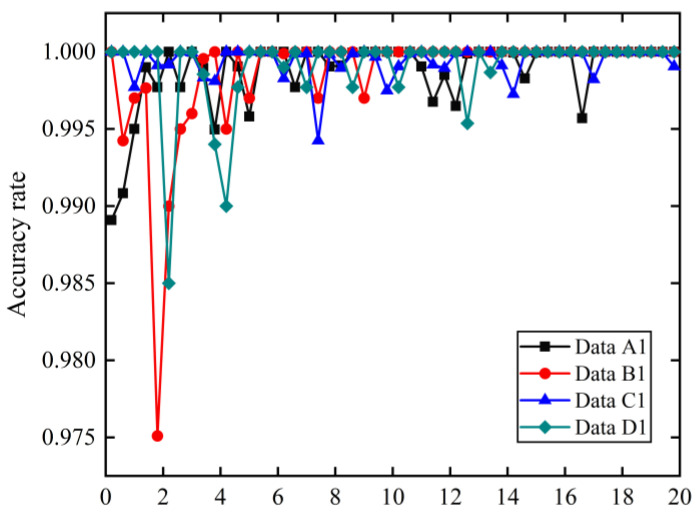
The accuracy curves of the proposed model training process.

Figure 16 and Table 10 show the accuracy curves of the proposed model training process and the final classification accuracy of the model under datasets *A*_1_–*D*_1_. To minimize specificity and chance, we repeated each experiment ten times and considered the average result of the ten experimental results as the accuracy of the model. From Figure 16 and Table 10, we can see that the model still achieves excellent diagnostic performance under different working conditions. The tested accuracies of the model under datasets *A*_1_–*D*_1_ are 99.97%, 99.78%%, 99.82%, and 99.69%, respectively. Thus, the two-dimensional grayscale images can still fully indicate different bearing states even under different operating conditions. It is also shown that the model has not only high fault diagnosis accuracy, but also good robustness for bearing fault diagnosis.

The vibration signals collected from mechanical bearings under complex working conditions incorporate with high power noise, which easily drowns the early fault information in strong background noise, thus making it impossible to achieve accurate fault detection. Therefore, to verify the noise robustness of the proposed method, signals with different signal-to-noise ratios were formed by adding additive Gaussian white noise (AWGN) with different standard deviations to the original vibration signals.

The signal-to-noise ratio is usually expressed in decibels as shown in Equation (22).

(22) $$SNR_{dB}=10\log_{10}\left(P_{\mathrm{signal}}/P_{\mathrm{noise}}\right)$$

**Figure 17:**
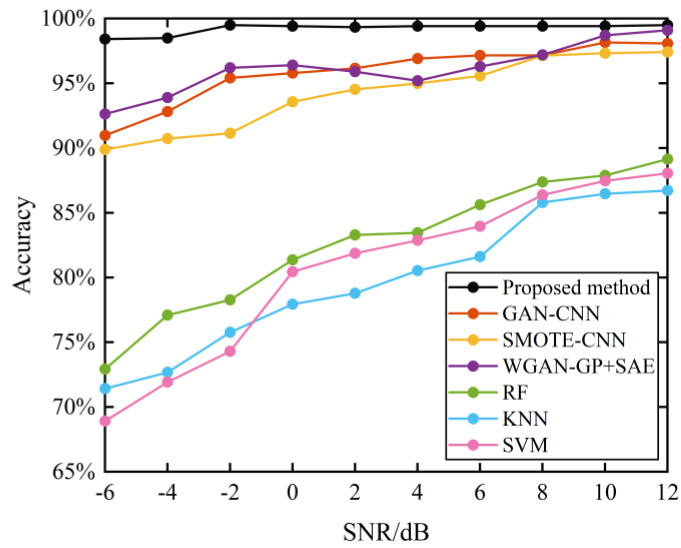
Comparison of accuracy testing under different noise environments.

Figure 17 shows the comparison of the diagnosis results of different algorithms under different noise environments. To avoid the effect of random factors on the experimental results, ten repetitive experiments were conducted for each test. From Figure 17, it can be seen that the diagnostic performance of all methods gradually augments with the increase of noise power, but the proposed method can achieve an accuracy of 98.264% under the robust noise pollution environment. The reason is that by changing the original one-dimensional vibration signal into two-dimensional grayscale images as the input samples for model training in the proposed method, sensitive features can be thoroughly mined from the complex original signal. At the same time, noise interference can be effectively suppressed.

Meanwhile, traditional machine learning (ML) such as *SVM* and KNN lead to poor diagnostic performance due to the scared capacity to restrain noise and unconcerned interference. Therefore, the proposed method has stronger robustness and superior diagnostic performance under solid ambient noise.

To contrapose the phenomenon of data imbalance in the fault diagnosis process, which leads to incomplete training of the deep network and the inability to completely fit the training sample distribution, ten imbalanced datasets with different data imbalance ratios were set to further assess the stability of the proposed method’s diagnostic performance.

The sample distributions of the ten imbalanced datasets with different imbalance ratios are shown in Table 11. In the ten imbalanced datasets, the ratios of normal samples and each genre of fault samples in the training dataset were set to 500:500, 500:450, 500:400, 500:350, 500:300, 500:250, 500:200, 500:150, 500:100, and 500:50, respectively, while the number of samples in the test dataset was set to 200.

**Figure 18:**
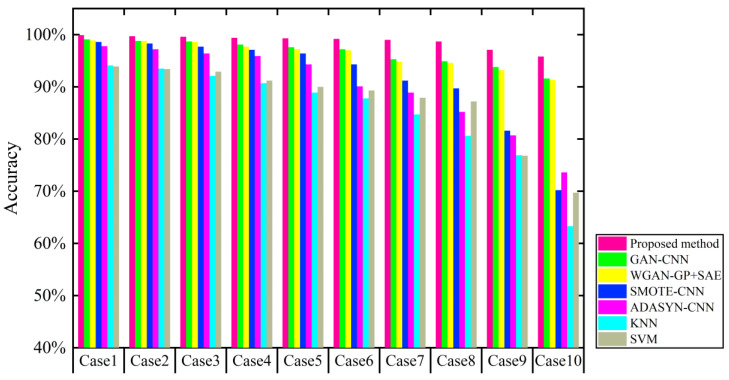
Contrast experiment under ten imbalanced cases.

To further verify this method’s validity under unbalanced data, we input the datasets under ten unbalanced states into the other five deep learning models as shown in Figure 18 and Table 12. The fault diagnosis precision rate of the proposed method under the first data distribution state is 99.9%, and the accuracy of the other six methods is 99.1%, 98.9%, 98.6%, 97.8%, 94.1%, and 93.9%, respectively.

When the training sample size under each fault category is reduced to half of the normal sample size, the fault diagnosis precision rate of the proposed method is much higher than that of the other six methods at 99.2%. The diagnostic performance of each diagnostic method decreases significantly as the data imbalance rate increases. When the imbalance rate reaches 10:1, the proposed method still shows good diagnostic performance. Therefore, although the fault identification accuracy of the proposed method tends to decrease with the intensification of the data imbalance rate, the method can still maintain a high diagnostic identification accuracy and has high diagnostic stability.

## 5. Conclusions and Future Work

In this research, an intelligent fault diagnosis method based on WGAN-GP and SECNN is proposed for rolling bearing fault diagnosis analysis under severe imbalance and distribution discrepancy of fault data. The method addresses the scenario of data imbalance under strong noise operation conditions. As an innovative application, the constructed model uses the signal-to-image conversion technique to convert the one-dimensional raw vibration signals into two-dimensional grayscale images, and the noise in the data is completely transformed into the grayscale, luminance, and other information in the images that are irrelevant to the image classification results, and the outstanding advantages of neural networks in two-dimensional image classification are fully reflected. WGAN-GP was used to generate more new data to overcome the distribution differences caused by data imbalance. Meanwhile, the attention mechanism was introduced, and a self-attentive convolutional neural network offline model was constructed to perform in-depth feature learning on the collected vibration signals, which can automatically and selectively enhance the useful feature mapping and reduce the redundant features on the convolutional channel.

The validity and meliorist of the method were verified by analyzing and discussing the benchmark data from CWRU and comparing it with other mainstream deep learning models. The experimental and computational results comparison shows that the method not only attains a diagnostic accuracy of more than 99.6% even under data imbalance and strong noise environment, but also has good generalization and robustness. The limitation of the proposed method is mainly focused on the sample generation of GANs, and in this study we generated more image samples similar to the original samples by GANs, and did not generate new image samples. However, there are many compound faults in the actual rolling bearing fault diagnosis process, so we cannot obtain the training samples under all compound fault modes. In the future work, we will further develop the signal-to-image transformation technique, deeply investigate the sample generation capability of GANs, and design a more suitable network.

## Figures and Tables

**Table 1:** The detailed parameters of deep groove rolling bearing.

Type	Value
Model	6205-2RSJEMSKF
Rolling element number (*z*)	9
Contact angle (θ)	0°
Rolling element diameter *d* (inches)	7.938
Pitch diameter *D* (inches)	1.537

**Table 2:** Description of the bearing dataset.

Identification Label	Sample Frequency	Fault Unit	Fault Diameter
0	12 k	Normal-Baseline	-
1	12 k	Drive End Fault-Ball	0.07
2	12 k	Drive End Fault-Ball	0.14
3	12 k	Drive End Fault-Ball	0.21
4	12 k	Drive End Fault-Inner	0.07
5	12 k	Drive End Fault-Inner	0.14
6	12 k	Drive End Fault-Inner	0.21
7	12 k	Drive End Fault-Outer	0.07
8	12 k	Drive End Fault-Outer	0.14
9	12 k	Drive End Fault-Outer	0.21

**Table 3:** Dataset partition and amount.

Label	*A*	*B* (*A* × 60%)	*C* (*A* × 40%)	*D* (WGAN-GP)	*E* (*B* + *D*)
	Raw Dataset	Training Dataset	Testing Dataset	Generated Dataset	Enhancement
0	24,313	14,588	9725	14,588	29,176
1	6065	3639	2426	3639	7278
2	6148	3689	2459	3689	7378
3	6126	3676	2450	3676	7352
4	6106	3664	2442	3664	7328
5	6081	3649	2432	3649	7298
6	6138	3683	2455	3683	7366
7	6098	3659	2439	3659	7318
8	6080	3648	2432	3648	7296
9	6135	3681	2454	3681	7362

**Table 4:** Gradient penalty factor λ set value comparison accuracy.

λ	1	5	10	15	20
Accuracy	0.938	0.973	0.994	0.936	0.957

**Table 5:** Fréchet distance and similarity of original and generated data.

		0	1	2	3	4	5	6	7	8	9
GAN	F	2.59	2.65	1.48	2.46	1.48	1.31	2.16	2.79	1.68	2.47
	S	0.39	0.38	0.68	0.41	0.68	0.76	0.46	0.36	0.60	0.40
WGAN	F	0.67	1.32	1.64	1.19	1.38	1.21	0.64	0.99	1.25	1.21
	S	1.49	0.76	0.61	0.84	0.72	0.83	1.56	1.01	0.80	0.83
WGAN-GP	F	0.79	1.31	0.99	0.38	0.84	0.43	1.19	0.67	1.48	0.64
	S	1.26	0.76	1.01	1.96	1.15	1.78	0.84	1.49	0.68	1.56

**Table 6:** The comparison results of different kernels and activation function.

	The Number of Convolution Kernels at				
Experimental Group	Each Convolution Layer			Activation Function	Accuracy
	L1	L2	L3		
1	8	16	32	*ReLU*	0.94
2	16	32	64	*ReLU*	0.96
3	32	64	128	*ReLU*	0.98
4	8	16	32	*Leaky ReLU*	0.96
5	16	32	64	*Leaky ReLU*	1.00
6	32	64	128	*Leaky ReLU*	0.99
7	8	16	32	*Tanh*	0.95
8	16	32	64	*Tanh*	0.99
9	32	64	128	*Tanh*	0.98

**Table 7:** Comparison of algorithm efficiency of the proposed model.

Number of Iterations	Training Time (min)	Testing Time (min)	Total Time (min)	Test Accuracy	λ
10,000	32	2	34	0.52	1.532
50,000	76	2	78	0.63	0.807
100,000	141	2	143	0.99	0.692
150,000	162	2	164	0.97	0.596
200,000	205	2	207	0.98	0.473

**Table 8:** Performance comparison of all approaches in testing samples.

Basic Algorithm	Hidden Layers	Classifier Type	Accuracy	Reference
CNN	4	*Softmax*	72.40%	[47]
Adaptive CNN	3	*Softmax*	87.94%	[48]
EMD-TDSF	6	*RVM*	83.21%	[49]
LMD-TDSF	6	*SVM*	93.27%	[49]
CNN-LSTM	3	*Softmax*	89.67%	[50]
CNN based on LeNet-5	8	*FC layer*	89.97%	[38]
K-means WGAN-GP	12	*RVM/SVM*	97.65%	[51]
GAN-CNN	9	*Softmax*	97.89%	[52]
WGAN-CNN	9	*Softmax*	99.1%	[53]
Proposed method	8	*Softmax*	99.6%	/

**Table 9:** Description of the bearing dataset.

Dataset	Bearing State	Training Set			Testing Set			Fault Diameter (mm)
		Samples	Speed (r·min^−1^)	Load (HP)	Samples	Speed (r·min^−1^)	Load (HP)	
*A*_1_	-/IF/BF/OF	11200	1797/1772/1750/1730	0/1/2/3	4200	1797/1772/1750/1730	0/1/2/3	-/0.18
*B*_1_	-/IF/BF/OF	8400	1797/1772/1750	0/1/2	2800	1730	3	-/0.18
*C*_1_	-/IF/BF/OF	5600	1797/1772	0/1	2800	1750	2	-/0.18
*D*_1_	-/IF_1_/IF_2_/IF_3_	5600	1797/1772	0/1	2800	1750	2	-/0.18/0.36/0.53

**Table 10:** Accuracy comparison under different working conditions.

Dataset	*A*_1_	*B*_1_	*C*_1_	*D*_1_	Average Accuracy
Accuracy	99.97%	99.78%	99.82%	99.69%	99.81%

**Table 11:** Description of the different imbalance ratios datasets.

Imbalanced	Number of Normal Condition		Number of Each Fault Condition	
Cases	Training Dataset	Testing Dataset	Training Dataset	Testing Dataset
Case 1	500	200	500	200
Case 2	500	200	450	200
Case 3	500	200	400	200
Case 4	500	200	350	200
Case 5	500	200	300	200
Case 6	500	200	250	200
Case 7	500	200	200	200
Case 8	500	200	150	200
Case 9	500	200	100	200
Case 10	500	200	50	200

**Table 12:** Contrast experiment under ten imbalanced cases.

Methods	Imbalanced Cases Accuracy										Average Accuracy
	Case1	Case2	Case3	Case4	Case5	Case6	Case7	Case8	Case9	Case10	
Proposed method	99.9%	99.7%	99.6%	99.4%	99.3%	99.2%	99%	98.7%	97.1%	95.8%	98.77%
GAN-CNN	99.1%	98.8%	98.7%	98.1%	97.6%	97.2%	95.3%	94.9%	93.8%	91.6%	96.51%
WGAN-GP + SAE	98.9%	98.8%	98.6%	97.7%	97.2%	97%	94.8%	94.6%	93.2%	91.3%	96.21%
SMOTE-CNN	98.6%	98.3%	97.7%	97.1%	96.4%	94.3%	91.2%	89.7%	81.6%	70.2%	91.51%
ADASYN-CNN	97.8%	97.2%	96.4%	95.9%	94.3%	90.1%	88.9%	85.2%	80.7%	73.6%	90.01%
KNN	94.1%	93.5%	92.1%	90.7%	88.9%	87.8%	84.7%	80.6%	76.9%	63.3%	85.26%
*SVM*	93.9%	93.4%	92.9%	91.2%	90%	89.3%	87.9%	87.2%	76.8%	69.7%	87.23%

## Data Availability

Not applicable.
